# Mutational Analysis of Anesthetic Binding Sites and Their Effects on GABA_A_ Receptor Activation and Modulation by Positive Allosteric Modulators of the α7 Nicotinic Receptor

**DOI:** 10.3390/biom13040698

**Published:** 2023-04-20

**Authors:** Spencer R. Pierce, Allison L. Germann, Sophia Q. Xu, Saumith L. Menon, Marcelo O. Ortells, Hugo R. Arias, Gustav Akk

**Affiliations:** 1 Department of Anesthesiology, Washington University School of Medicine, St. Louis, MO 63110, USA; 2 Facultad de Medicina, Universidad de Morón, CONICET, Morón 1708, Argentina; 3 Department of Pharmacology and Physiology, Oklahoma State University College of Osteopathic Medicine, Tahlequah, OK 74464, USA; 4 The Taylor Family Institute for Innovative Psychiatric Research, Washington University School of Medicine, St. Louis, MO 63110, USA

**Keywords:** GABA_A_ receptor, α7 nicotinic receptor, modulation, potentiator, allostery

## Abstract

The positive allosteric modulators (PAMs) of the α7 nicotinic receptor *N*-(5-Cl-2-hydroxyphenyl)-*N*′-[2-Cl-5-(trifluoromethyl)phenyl]-urea (NS-1738) and (*E*)-3-(furan-2-yl)-*N*-(*p*-tolyl)-acrylamide (PAM-2) potentiate the α1β2γ2L GABA_A_ receptor through interactions with the classic anesthetic binding sites located at intersubunit interfaces in the transmembrane domain of the receptor. In the present study, we employed mutational analysis to investigate in detail the involvement and contributions made by the individual intersubunit interfaces to receptor modulation by NS-1738 and PAM-2. We show that mutations to each of the anesthetic-binding intersubunit interfaces (β+/α−, α+/β−, and γ+/β−), as well as the orphan α+/γ− interface, modify receptor potentiation by NS-1738 and PAM-2. Furthermore, mutations to any single interface can fully abolish potentiation by the α7-PAMs. The findings are discussed in the context of energetic additivity and interactions between the individual binding sites.

## 1. Introduction

The γ-aminobutyric acid type A (GABA_A_) receptor is a Cl^−^-permeable transmitter-gated ion channel. Although found in select non-neural tissue, it is mostly, and widely, expressed in neurons where its activation by synaptically released or ambient GABA leads to cellular hyperpolarization or dampening of the effects of excitatory ion channels. The receptor is also activated by 2-aminoethanesulfonic acid (taurine), while various neurosteroids can potentiate (e.g., allopregnanolone) or inhibit (e.g., pregnenolone sulfate) its function. The GABA_A_ receptor is a target for numerous anxiogenics, sedatives, and anesthetics used in clinical practice. Studies of the mechanisms of receptor activation and modulation, particularly in the simultaneous presence of multiple active compounds, are crucial to our understanding of the physiological and pathophysiological roles of the GABA_A_ receptor.

The common α1β2γ2L subtype of the GABA_A_ receptor contains two transmitter-binding (orthosteric) sites in the extracellular domain at the two β+/α− intersubunit interfaces [1,2]. There is a homologous site at the α+/γ− interface through which benzodiazepines act on the GABA_A_ receptor [3]. In the transmembrane domain, the receptor contains several interfacial and intrasubunit sites for anesthetics, benzodiazepines, and neurosteroids [4,5,6]. Co-application of an allosteric agonist potentiates the response to subsaturating GABA or to another allosteric agonist (e.g., [7,8,9,10]). In the “co-agonist model” [11,12], each activator is postulated to make an independent and additive energetic contribution towards the stabilization of the active state. This enables a model-based prediction of the response amplitude for agonist combinations if the properties of each agonist are known. The co-agonist model-based predictions work, within limits of experimental error, for the combinations of GABA plus propofol, etomidate, benzodiazepines, or neurosteroids [13,14,15,16,17,18]. On the other hand, discrepancies between experimental observations and predictions based on energetic additivity have been observed for several combinations of anesthetic drugs including the combinations of etomidate plus propofol and etomidate plus barbiturate [10]. Furthermore, mutations to individual anesthetic binding sites have been shown to modify the potentiating effects of the anesthetics binding at that site as well as those binding to homologous sites at other interfaces [19]. This has been interpreted as an allosteric linkage between the sites.

**Figure 1:**
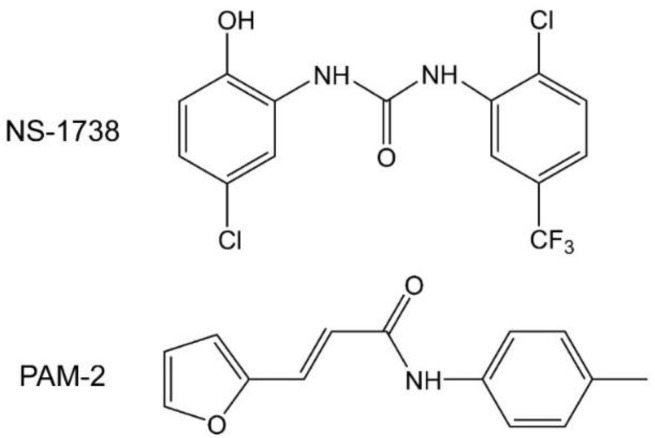
Structural formulas of *N*-(5-Cl-2-hydroxyphenyl)-*N*′-[2-Cl-5-(trifluoromethyl)phenyl]-urea (NS-1738) and (*E*)-3-(furan-2-yl)-*N*-(*p*-tolyl)-acrylamide (PAM-2).

The anesthetic binding sites are located at intersubunit interfaces in cavities formed by the second and third transmembrane domains (TM2 and TM3) of the subunit contributing to the “+” side of the interface and the first transmembrane domain (TM1) of the subunit contributing to the “−” side of the interface. The sedative anesthetic etomidate, for example, binds to the β+/α− interface, whereas a barbiturate derivative binds with high affinity to the α+/β− and γ+/β− interfaces [20,21]. We recently showed that type I and type II positive allosteric modulators (PAMs) of the α7 nicotinic receptor *N*-(5-Cl-2-hydroxyphenyl)-*N*′-[2-Cl-5-(trifluoromethyl)phenyl]-urea (NS-1738) and (*E*)-3-(furan-2-yl)-*N*-(*p*-tolyl)-acrylamide (PAM-2) (Figure 1) potentiate the α1β2γ2L GABA_A_ receptor through interactions with anesthetic binding sites [22]. Here, we have employed mutational analysis to investigate in detail the involvement and contributions made by the individual intersubunit interfaces to receptor modulation by NS-1738 and PAM-2. A major observation is that mutations to any single interface can fully abolish potentiation by the α7-PAMs, which is indicative of a lack of independently acting sites. The findings are discussed in the context of energetic additivity and allosteric interactions between individual binding sites.

## 2. Materials and Methods

### 2.1. Molecular Modeling

Modeling of the α1β2γ2 GABA_A_ receptor was done using the Prime module of the Schrödinger Suite Release 2020-3 (Schrödinger, LLC, New York, NY, USA). For the template, we used the human α1β2γ2 GABA_A_R structure (PDB: 6X3T; [23]). The molecular structures of NS-1738 and PAM-2 were prepared and evaluated for their ionization states at pH 7.4 using 2D Sketcher and LigPrep within Schrödinger Maestro (Maestro Version 12.5.139). Each ligand was docked to the α1β2γ2 GABA_A_ receptor model using QuickVina-W [24]. The exhaustiveness parameter was set to 300. Ten docking runs, each producing twenty poses, were performed under the same conditions. The poses with more negative theoretical binding energy values, indicating higher theoretical binding affinities, were stored. The final selected docked conformers were further analyzed using molecular dynamics (MD) to determine their stability and behavior within the binding site. Membrane building (dipalmitoylphosphatidylcholine and cholesterol), complex solvation, and ionization were carried out using CHARMM-GUI [25]. To determine the stability of the selected poses within their predicted docking sites, MD simulations of 100 ns were performed using the program NAMD, CHARMM force field [26], and CHARMM-GUI NAMD input generator with the PETE supercomputer at the High Performance Computing Center (Oklahoma State University Center for Health Sciences, Tulsa, OK, USA).

### 2.2. Receptors, Expression, and Electrophysiology

The cDNAs for rat α1 (Genbank accession number NM_183326), β2 (NM_012957), and γ2L (NM_183327) subunits in the pcDNA3 vector were linearized with the XbaI (NEB Laboratories, Ipswich, MA, USA) restriction enzyme. The cRNAs were synthesized from linearized cDNA using mMessage mMachine (Life Technologies, Grand Island, NY, USA). Mutant clones were purchased from Twist Bioscience (South San Francisco, CA, USA).

Wild-type and mutant GABA_A_Rs were expressed in *Xenopus laevis* oocytes. The oocytes were purchased from Xenopus 1 (Dexter, MI, USA) as quarter ovaries. To remove the follicular membrane, ovaries were incubated in 2% *w*/*v* (mg/mL) Collagenase A (Sigma-Aldrich, St. Louis, MO, USA) solubilized in ND96 solution (96 mM NaCl, 2 mM KCl, 1.8 mM CaCl_2_, 1 mM MgCl_2_, 5 mM HEPES; pH 7.4) with supplements (2.5 mM Na pyruvate, 100 U/mL penicillin, 100 μg/mL streptomycin and 50 μg/mL gentamycin) at 37 °C with shaking at 250 RPM for 30 min. Following a subsequent 3–4 h incubation at 15 °C, the oocytes were injected with a total of 3.5 ng of cRNA (0.5 ng:0.5 ng:2.5 ng, α1:β2:γ2L) per oocyte. The injected oocytes were incubated in ND96 with supplements for 1–2 days before commencing electrophysiological recordings.

Two-electrode voltage-clamp recordings were conducted as described in detail previously [27,28]. The oocytes were placed in a recording chamber (RC-1Z, Warner Instruments, Hamden, CT, USA) and clamped at −60 mV. Borosilicate glass capillaries (G120F-4, Warner Instruments) were used as voltage and current electrodes. When filled with 3 M KCl, the electrodes had typical resistances of ~1 MΩ. Solutions were gravity-applied from glass syringes and switched manually using 4-port bulkhead switching valves and medium-pressure 6-port bulkhead valves (IDEX Health and Science, Rohnert Park, CA, USA). The current responses were amplified with an OC-725C amplifier (Warner Instruments), digitized with a Digidata 1200 series digitizer (Molecular Devices, San Jose, CA, USA), and stored using pClamp (Molecular Devices).

### 2.3. Cysteine-Modification Experiments

Cysteine-modification experiments were done by exposing a wild-type (control) or a cysteine-mutated receptor to *p*-chloromercuribenzoic acid (pCMB). Successful modification of a cysteine residue was deduced from irreversible alteration of receptor function following the application of pCMB [29,30,31]. The pCMB concentration was 25 µM and the application duration was 30 s. pCMB was applied in the presence of 1 mM GABA, and all drug applications were followed by a 4–5 min wash in ND96. In modification-protection experiments, the cells were exposed to the protecting agent (NS-1738 or PAM-2) for 60 s, followed by a 30 s application of pCMB + GABA + protective agent.

### 2.4. Functional Studies and Data Analysis

The modulatory effects of α7-PAMs were examined under two experimental protocols. In cases where the constitutive probability of being in the active state (P_A,constitutive_) was low (<0.02), modulation was established by coapplying α7-PAM during a steady-state response to a low concentration (P_A_ < 0.10) of GABA. The P_A,low GABA_ was not corrected for P_A,constitutive_ when the latter was less than 0.01. Drug effects were initially quantified by calculating fold-potentiation from the ratio of the peak response to GABA+α7-PAM to the steady-state response to GABA immediately before the application of the modulator. In cases where P_A,constitutive_ was high (>0.02), the α7-PAMs were applied in the absence of GABA, and drug effects were calculated from the estimated probability of being in the active state (P_A,α7-PAM_) as P_A,α7-PAM_/P_A,constitutive_. P_A,constitutive_ was estimated by comparing the effects on holding current by 200 µM picrotoxin, which was assumed to block all receptors (P_A_ = 0), and 1 mM GABA + 50 µM propofol, which was assumed to activate all receptors (P_A_ = 1) [32].

For mechanistic analysis of receptor modulation by α7-PAMs, the data were analyzed in the framework of the two-state (Resting-Active) cyclic model [11,12]. The state function of the model is as follows:

(1) $$P_{A,\alpha 7\text{-}\mathrm{PAM}}=\frac{1}{1+((1-P_{A,\mathrm{background}})/P_{A,\mathrm{background}}){\left[\frac{1+[\alpha 7\text{-}\mathrm{PAM}]/K_{R,\alpha 7\text{-}\mathrm{PAM}}}{1+[\alpha 7\text{-}\mathrm{PAM}]/(K_{R,\alpha 7\text{-}\mathrm{PAM}}c_{\alpha 7\text{-}\mathrm{PAM}})}\right]}^{N_{\alpha 7\text{-}\mathrm{PAM}}}}$$

where P_A,α7-PAM_ and P_A,background_ are the P_A_ in the presence and absence, respectively, of the compound studied. K_R, α7-PAM_ is the equilibrium dissociation constant of the compound in the resting receptor, *c*_α7-PAM_ is the ratio of the equilibrium dissociation constant of the compound in the active receptor to K_R, α7-PAM_, [α7-PAM] is the concentration of the compound, and N _α7-PAM_ is the number of imposed binding sites (by convention, 2).

In all experiments, NS-1738 and PAM-2 were tested at 50 µM, which is a near-saturating concentration in the α1β2γ2L receptor [22]. The parameter *c*_α7-PAM_ that reflects gating efficacy (ratio of equilibrium dissociation constants in the active and resting receptors) was calculated as follows:

(2) $$c_{\alpha 7\text{-}\mathrm{PAM}}=\sqrt[{1/N_{\alpha 7\text{-}\mathrm{PAM}}}]{\frac{1/P_{A,\alpha 7\text{-}\mathrm{PAM}}-1}{(1-P_{A,\mathrm{background}})/P_{A,\mathrm{background}}}}$$

Free energy change (ΔG) was calculated from *c*_α7-PAM_ as ΔG = NRT × ln(*c*_α7-PAM_). The gating efficacies of the tested anesthetic compounds were estimated by analogous calculations.

**Figure 2:**
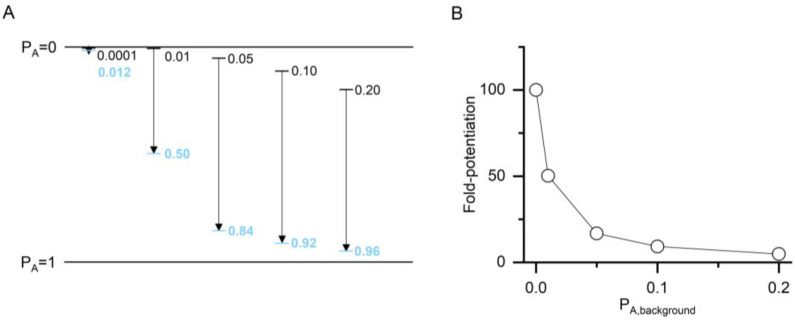
The relationship between apparent potentiation and the value of *c*_compound_. (**A**) The plot shows imposed P_A,background_ (black lines and numbers) and calculated P_A,compound_ (blue lines and numbers) for a hypothetical compound with two binding sites (N = 2) and a *c*_compound_ of 0.100 (ΔG = −2.72 kcal/mol). The calculations were done at a saturating concentration (1000× of K_resting_) and thus reflect maximal effects. Higher P_A_ is shown downward for consistency with the direction of current flow in electrophysiological recordings from GABA_A_ receptors. (**B**) The graph shows fold-potentiation at different P_A,background_ calculated from the data in panel A. The compound has a larger apparent potentiating effect when measured at low P_A,background_.

The value of *c*_compound_ and, consequently, that of ΔG is a measure of the ability of a compound to increase the P_A_ of a response. It non-linearly expresses the difference between the P_A_ of the control response (P_A,background_) and the P_A_ of the response in the presence of a modulatory compound (P_A,compound_). The relationship between the background activity and *c*_compound_ is illustrated in Figure 2. The apparent potentiating effect (the calculated fold-potentiation) of a compound is dependent on the P_A_ value of the background, i.e., control response, thus introducing an error if fold-potentiation is compared at different P_A,background_. The use of *c*_compound_ to compare effects negates this. The nominal value of *c*_compound_, however, depends on the number of postulated binding sites for the compound. This precludes direct comparison of *c* values for compounds with differing numbers of binding sites. ΔG, on the other hand, reflects the total stabilization energy provided by a compound, and its nominal value is independent of the number of binding sites.

### 2.5. Materials

Salts used in ND96, HEPES, GABA, propofol, and 5β-pregnan-3α-ol-20-one (3α5βP) were purchased from Sigma-Aldrich (St. Louis, MO, USA). Etomidate was purchased from Toronto Research Chemicals (Toronto, ON, Canada). NS-1738 was obtained from Cayman Chemical (Ann Arbor, MI, USA) and Adooq Bioscience (Irvine, CA, USA). PAM-2 was synthesized as described previously [33]. A stock solution of 500 mM GABA in ND96 was stored at 4 °C. All other stock solutions were made in dimethyl sulfoxide (Sigma-Aldrich) with 200 mM propofol and 20 mM 3α5βP stocks stored at room temperature and 200 mM etomidate, 100 mM NS-1738, and 100 mM PAM-2 stocks stored at −20 °C. Final dilutions were made on the day of the experiment. 

## 3. Results

### 3.1. Molecular Docking and Molecular Dynamics Indicate Binding Sites for α7-PAMs at Intersubunit Interfaces in the Transmembrane Domain

**Figure 3:**
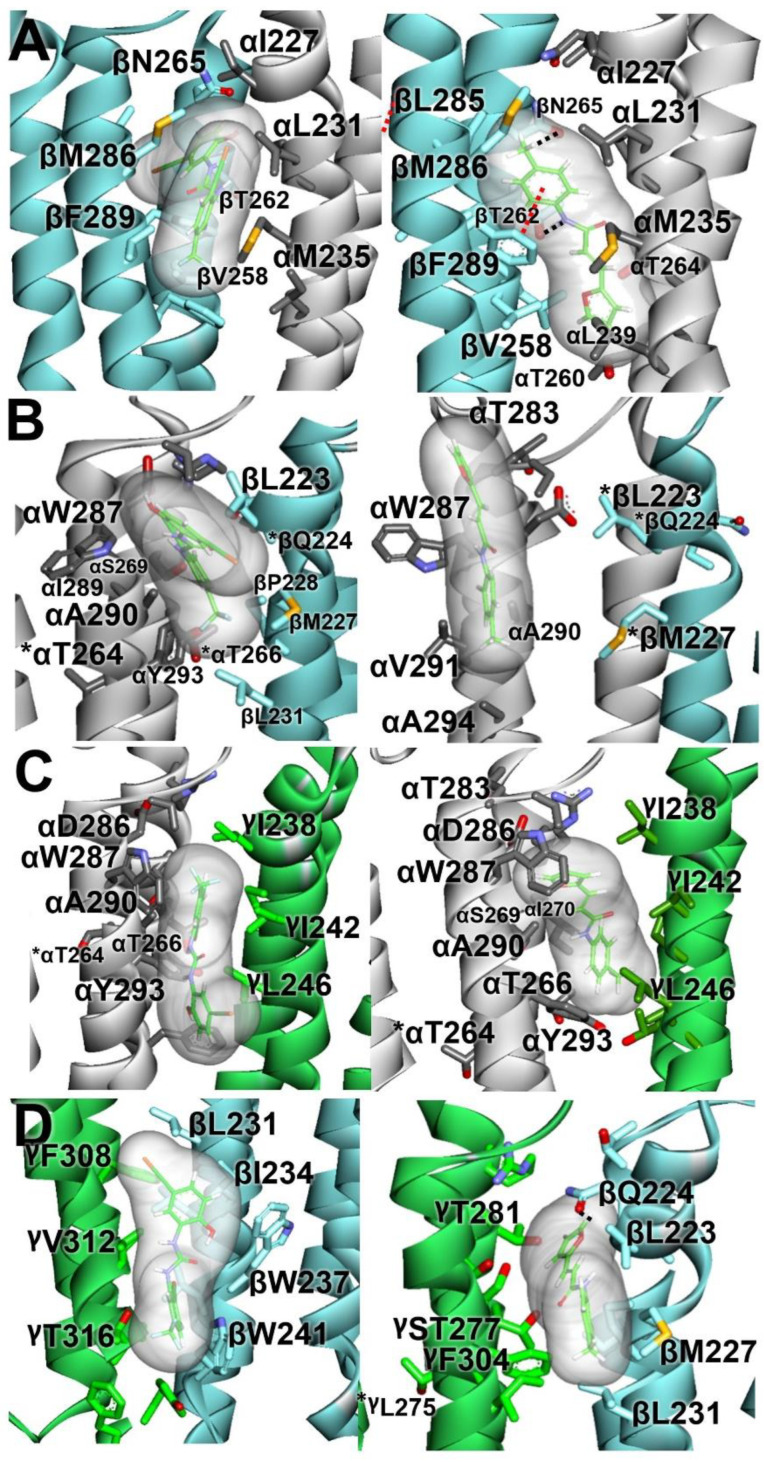
Molecular docking of NS-1738 (left panels) and PAM-2 (right panels) to the α1β2γ2 GABA_A_ receptor model. Stable docking sites are shown at the β+/α− (**A**), α+/β− (**B**), α+/γ− (**C**), and γ+/β− (**D**) interfaces. Subunits (α, white; β, light-blue; and γ, green) are represented as ribbons, while ligands are represented as thin sticks surrounded by their molecular surfaces, colored by atoms, with carbon atoms in green. The interacting residues are represented by thick sticks, colored by atoms, with H atoms omitted for clarity. Black dotted lines represent hydrogen bonds. Red dotted lines represent π–π interactions. * Residues were tested with mutational analysis but were not predicted to interact with the ligand.

The docking experiments were based on the structure of the human α1β2γ2 receptor (PDB: 6X3T) [23]. In the APO 6X3T structure, both NS-1738 and PAM-2 were able to dock in the anesthetic binding sites at the β+/α−, α+/β−, and γ+/β− interfaces. In addition, the compounds were shown to bind at a homologous site at the α+/γ− interface. The structure figures with docked α7-PAMs are given in Figure 3. The corresponding PDB files are provided in the Supplementary Materials. The amino acid sequences of the human and rat α1 subunits differ. The human subunit has one extra residue (a leucine in position 4 in the mature subunit) in the amino-terminal region but is otherwise identical to rat α1. For convenience and consistency with electrophysiological data, we used the rat numbering when discussing specific residues in the α1 subunit (hence, the true numbering in human α1 = the provided rat numbering + 1).

At the β+/α− interface, NS-1738 and PAM-2 are sandwiched, nearly in parallel with the α-helical transmembrane segments, in the cavity between β-TM3, β-TM2, and α-TM1. The 2-hydroxyl group of NS-1738 and the tolyl group of PAM-2 are oriented towards the extracellular side of the membrane. The β2(F289) residue contributes to carbonyl–aromatic (CO-π) interaction with the carbonyl group of NS-1738, whereas, one α-helical turn-up, β2(M286) borders the chloro-hydroxyphenyl ring. At the “−” side of the interface, the α1(M235) residue abuts the trifluoromethylphenyl group of NS-1738, and α1(L231) and α1(I227) further up in α1-TM1 border the amine and phenylhydroxyl groups. The tolyl group of PAM-2 is oriented between the β2(M286), β2(F289), and α1(I227) residues. The carbonyl of acrylamide points towards α1(T236).

At the γ+/β− interface, NS-1738 is positioned at the protein–lipid interface between γ2-TM3 and β2-TM1. NS-1738 is oriented in parallel with the transmembrane helices, with the trifluoro and phenyl groups positioned between γ2(T316) and β2(W241), and pointed towards the cytosolic side of the membrane. The chloro-hydroxyphenyl ring is located between γ2-TM3 and β2-TM1, near γ2(F308) and β2(I234). In contrast, PAM-2 lies, nearly perpendicular to the transmembrane helices, in a cavity between γ-TM2, γ-TM3, and β-TM1. The furan group rests between γ2(T281) and β2(Q224) in γ2-TM2 and β2-TM1, respectively, whereas the tolyl group of PAM-2 is sandwiched between γ2(F304) and β2(M227).

At the α+/β− interface, the NS-1738 is positioned between α-TM3 and γ-TM1. The trifluoro group points towards the cytosolic side of the membrane, surrounded by α1(Y293) from the “+” side and β2(M227) and β2(L231) from the “−” side of the interface. The oxygen of the chloro-hydroxyphenyl ring is within 5 Å of α1(W287). PAM-2 is oriented in parallel with the transmembrane domains with its tolyl group pointed towards the extracellular side. The molecule clinches to α-TM3 with the carbonyl of acrylamide within 4 Å of α1(A290).

At the α+/γ− interface, NS-1738 is placed between the α-TM3 and γ-TM1 domains. It is oriented in parallel with the α-helical transmembrane segments, with the trifluoro group pointing towards the extracellular side of the membrane and located within 3.4 Å of α1(A290). The 2-hydroxyl oxygen of NS-1738 borders α1(A294), one α-helical turn down towards the cytosolic side of the membrane. At the “−” side, γ2(I242) and γ2(L246) position near the trifluoro and carbonyl groups, respectively. Like at the γ+/β− interface, PAM-2 is positioned nearly perpendicular to the transmembrane segments. The furan group oxygen is ~3 Å from α1(A290) and less than 5 Å from α1(W287). The carbonyl group of acrylamide is within 5 Å of γ2(I242) and γ2(L246) residues, whereas the methyl group of tolyl ring points to γ2(T275) (the 10′ residue in γ-TM2).

### 3.2. Mutations to the β+/α− Interface Affect Receptor Activation and Potentiation by α7-PAMs

**Figure 4:**
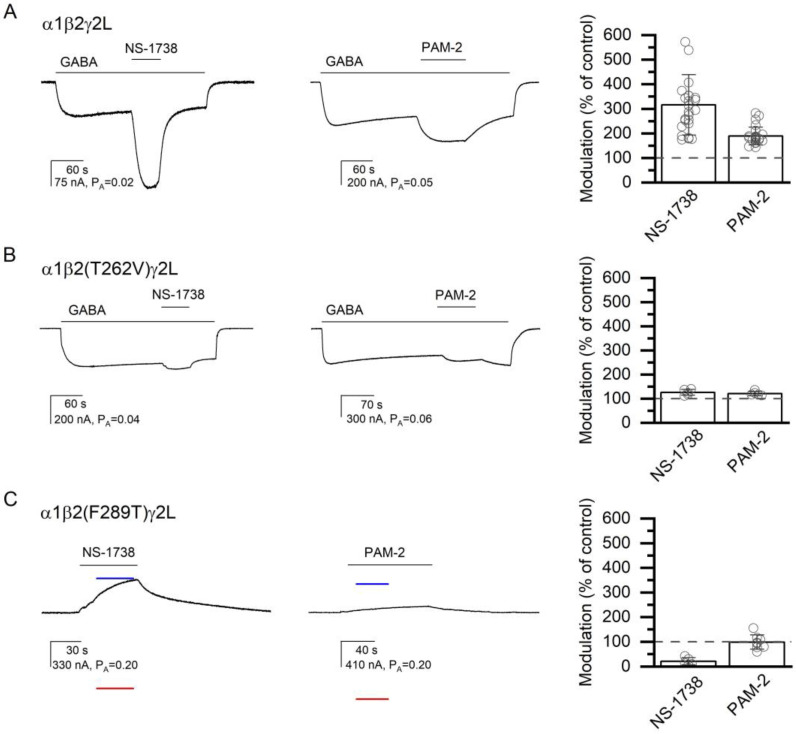
The modulatory effects of NS-1738 and PAM-2. The current traces show the potentiating effects of 50 µM NS-1738 or PAM-2 in the α1β2γ2L receptor activated by 2 µM GABA (**A**), or in the α1β2(T262V)γ2L receptor activated by 4 µM GABA (**B**). Panel (**C**) shows direct activation by NS-1738 or PAM-2 of the constitutively active α1β2(F289T)γ2L receptor. The blue and red lines show the current levels in the same cell in the presence of 200 µM picrotoxin (assumed P_A_ = 0) or 1 mM GABA + 50 µM propofol (assumed P_A_ = 1), respectively. The modulatory effects are summarized in the column graphs. In (**A**,**B**), modulation is calculated as the amplitude of the peak response to GABA + α7-PAM divided by the amplitude of the response to GABA alone at the time of peak response to GABA + α7-PAM. In (**C**), modulation is calculated as the ratio of the estimated P_A_ in the presence and absence of α7-PAM. Drug application durations are given with horizontal lines.

In the wild-type α1β2γ2L receptor, 50 µM NS-1738 potentiated the responses to low GABA (P_A_ = 0.05 ± 0.03, *n* = 25) to 316 ± 123% of the control. The application of 50 µM PAM-2 potentiated the response to GABA (P_A_ = 0.06 ± 0.03, *n* = 25) to 189 ± 36% of the control. With N_α7-PAM_ constrained to two, the calculated *c*_50 µM NS-1738_ is 0.561 ± 0.106 (ΔG = −0.70 ± 0.23 kcal/mol), and the calculated *c*_50 µM PAM-2_ is 0.721 ± 0.061 (ΔG = −0.39 ± 0.10 kcal/mol). Sample current traces showing potentiation of the wild-type receptor by the α7-PAMs are given in Figure 4A, and the data are summarized in Table 1. 

At the β+/α− interface, we tested the effects of mutations to the β2(V258), β2(T262), β2(F289), α1(I227), and α1(M235) residues. In the β2(V258M)-containing receptor, NS-1738 potentiated the response to GABA (P_A_ = 0.09 ± 0.02, *n* = 5) to 720% of the control (S.D. and numbers of oocytes are given in Table 1). Application of PAM-2 enhanced the response to GABA (P_A_ = 0.10 ± 0.04, *n* = 5) to 301% of the control. Replacement of the polar threonine with hydrophobic valine at position 262 in the β2 subunit (β2(T262V)) reduced the potentiating actions of both α7-PAMs. Co-application of 50 µM NS-1738 increased the response to 3–4 µM GABA (P_A_ = 0.08 ± 0.03; *n* = 5) to 126% of the control (P_A,50 µM NS-1738_ = 0.10 ± 0.03). Exposure to 50 µM PAM-2 increased the response to GABA (P_A_ = 0.05 ± 0.03; *n* = 5) to 121% of the control (P_A,50 µM PAM-2_ = 0.06 ± 0.04). Both values are statistically significantly lower than the magnitude of potentiation observed in the wild-type α1β2γ2L receptor. The summary of the effects of mutations on GABA_A_ receptor potentiation or direct activation by α7 PAMs on mutant receptors is provided in Table 1. Sample current traces for the α1β2(T262V)γ2L receptor are given in Figure 4B.

The β2(F289A) mutant receptor exhibited a considerable level of constitutive activity (P_A,const_ = 0.025 ± 0.019, *n* = 25). The direct-activating and potentiating actions of NS-1738 and PAM-2 were abolished in the mutant receptor. In the absence of GABA, exposure to NS-1738 or PAM-2 reduced P_A,const_ from 0.035 ± 0.030 (n = 5) to 0.016 ± 0.009 (74% of the control) or from 0.035 ± 0.021 (*n* = 5) to 0.029 ± 0.013 (87% of the control), respectively. In the presence of 0.1 µM GABA, the application of NS-1738 reduced the P_A_ from 0.06 ± 0.02 (*n* = 5) to 0.02 ± 0.01 (29% of the control), and the application of PAM-2 decreased P_A_ from 0.06 ± 0.04 (*n* = 5) to 0.04 ± 0.03 (81% of the control). 

A threonine substitution at β2(F289) (P_A,const_ = 0.25 ± 0.06, *n* = 13) similarly reduced the activating actions of α7-PAMs. The application of NS-1738 reduced the P_A_ from 0.28 ± 0.05 (*n* = 5) to 0.04 ± 0.05 (17% of the control). Exposure to PAM-2 had a minimal effect; the P_A_ was 0.23 ± 0.06 (*n* = 8) in the absence and 0.22 ± 0.05 (98% of the control) in the presence of the compound. Sample current traces are given in Figure 4C. Thus, in relative terms, amino acid substitutions at β2(F289) had greater effects on the actions of NS-1738 for which the mutations are predicted to lead to the loss of the carbonyl–aromatic (CO-π) interaction, compared to the actions of PAM-2 that makes π–π interactions with β2(F289). 

The introduction of the β2(V289M) mutation increased the potentiating effects of α7-PAMs (Table 1). The mechanism of this is unclear but is likely to be indirect; the residue is >9Å from the trifluoro group of NS-1738 although only ~3Å from the furan oxygen in PAM-2. 

At the “−” side of the β+/α− interface, the α1(I227W) and α1(M235W) mutations minimally reduced potentiation or direct activation by α7-PAMs. In the α1(I227W) mutant, exposure to NS-1738 or PAM-2 increased the response to 0.05–0.08 µM GABA (P_A_ = 0.07 ± 0.03) to 207% of the control (indistinguishable from α1β2γ2L) or 135% (*p* < 0.05 vs. α1β2γ2L), respectively. The α1(M235W) mutant was constitutively active (P_A,const_ = 0.048 ± 0.025, total *n* = 18). Application of NS-1738 increased the P_A_ from 0.053 ± 0.023 (*n* = 5) to 0.096 ± 0.027 (196% of the control), and application of PAM-2 increased the P_A_ from 0.042 ± 0.026 (*n* = 5) to 0.071 ± 0.040 (174% of the control). Neither differed from the potentiation observed in α1β2γ2L. We also tested the effect of the α1(L231C) mutation on the β+/α− interface. Potentiation by α7-PAMs was not affected by the mutation. These results are described in detail in the next section. In sum, we infer from the mutational-functional experiments that the β+/α− interface participates in the potentiation of the ternary α1β2γ2L receptor by α7-PAMs.

### 3.3. Cysteine-Modification Experiments Indicate the Binding of α7-PAMs at the β+/α− Interface

To verify the binding of α7-PAMs in the anesthetic binding site at the β+/α− interface, we used substituted cysteine modification-protection (SCAMP). In this approach, a receptor containing a cysteine substitution in the region of interest is exposed to a cysteine-modifying agent whose effect on receptor function is compared in the absence and presence of a protective drug [29,30]. Here, we examined the protective effects of NS-1738 and PAM-2 on the functional effect of *p*-chloromercuribenzoic acid (pCMB) on receptor function. In all experiments, pCMB was co-applied with 1 mM GABA.

The experiments were done on the α1(L231C)β2γ2L receptor. We employed two related experimental protocols. In the first, we compared the ratio of low (3 µM) and high (1 mM) GABA, before and after exposure to pCMB, which was applied alone (control) or in the presence of NS-1738 or PAM-2. In seven cells, the ratio of low-to-high GABA was 0.12 ± 0.04 before and 0.57 ± 0.15 (a 5.0 ± 1.8-fold increase) after a 30 s exposure to 25 µM pCMB. The increase in the GABA response ratio was due to an increase in the amplitude of the response to low GABA. We interpret this observation as labeling of the α1(L231C) residue with pCMB modifying receptor function.

**Figure 5:**
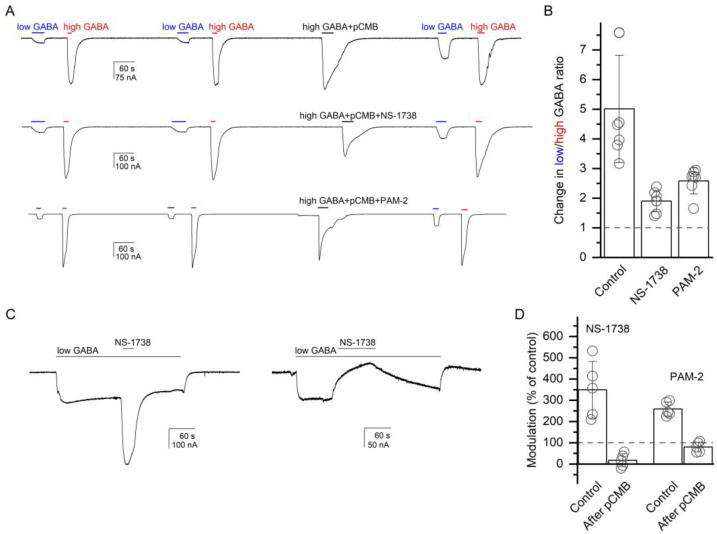
Chemical modification of the α1(L231C) residue at the β+/α− interface modifies receptor activation by GABA and modulation by α7-PAMs. (**A**) Exposure to pCMB (30 s, 25 µM) increases the ratio of responses to low (3 µM) and high (1 mM) GABA in the α1(L231C)β2γ2L receptor. The pCMB-induced increase in the low-to-high GABA ratio is reduced when pCMB is co-applied with 50 µM NS-1738 (middle trace) or PAM-2 (bottom trace). The data are summarized in the column graph (**B**). We infer that chemical modification of the α1(L231C) residue modifies responses to the transmitter and that co-application of an α7-PAM protects against labeling with pCMB. (**C**) Exposure to 50 µM NS-1738 potentiates the response to 4 µM GABA (P_A_ = 0.03) in the α1(L231C)β2γ2L receptor (left trace). NS-1738 inhibits the response to 0.05 µM GABA (P_A_ = 0.01) following a 30 s exposure to 25 µM pCMB. The traces are from different oocytes. Drug application durations are given with horizontal lines. The data for NS-1738 and PAM-2 are summarized in the column graph (**D**). We infer that pCMB-labeling of the α1(L231C) residue abolishes receptor potentiation by α7-PAMs.

In the presence of 50 µM NS-1738, the application of pCMB increased the low-to-high GABA ratio from 0.12 ± 0.04 (*n* = 7) to only 0.23 ± 0.09 (a 1.9 ± 0.4-fold increase). Additionally, in the presence of 50 µM PAM-2, exposure to pCMB enhanced the low-to-high GABA ratio from 0.12 ± 0.06 (*n* = 7) to 0.31 ± 0.16 (a 2.6 ± 0.4-fold increase). Sample current traces are given in Figure 5A. There was a statistically significant difference between pCMB applied in the absence or presence of α7-PAMs as determined by one-way ANOVA (*F*(2,18) = 24.25, *p* < 0.001). A Bonferroni post-hoc test revealed that the effect of pCMB in the absence of α7-PAMs was statistically significantly different compared to that in the presence of NS-1738 or PAM-2 (*p* < 0.001 for each comparison). We infer that NS-1738 and PAM-2 can protect against pCMB-induced labeling of the α1(L231C) residue.

In the second experiment, we compared the potentiation of the α1(L231C)β2γ2L receptor by α7-PAMs before and after exposure to pCMB. We reasoned that if NS-1738 and PAM-2 potentiate the GABA_A_ receptor through interactions with the β+/α− interface then labeling of α1(L231C) with pCMB may occlude the site and reduce α7-PAM-induced potentiation. Application of 50 µM NS-1738 potentiated the response to low (3–4 µM) GABA (P_A_ = 0.06 ± 0.02, *n* = 5) to 349 ± 133% of the control. Following a 30 s exposure to 50 µM pCMB, NS-1738 inhibited the response to low GABA to 17 ± 31% (*n* = 5) of the control. As noted above and illustrated in Figure 5A, exposure to pCMB leads to an increase in the relative response to low GABA. Accordingly, GABA concentration in this experiment was lowered to 0.05 µM, which generated a response with P_A_ of 0.13 ± 0.09 following exposure to pCMB. PAM-2 potentiated the response to 2 µM GABA (P_A_ = 0.10 ± 0.02, *n* = 5) to 197 ± 19% of the control. Following exposure to pCMB, exposure to PAM-2 resulted in a reduction (80 ± 23% of the control, *n* = 5) in the current response to 0.05 µM GABA (P_A_ = 0.13 ± 0.11). We conclude that the β+/α− interface mediates receptor potentiation by NS-1738 and PAM-2, and that occlusion of the site at this interface by pCMB abolishes potentiation.

In control experiments on the wild-type α1β2γ2L receptor, a 30 s exposure to 100 µM pCMB was without effect on the P_A_ of the response to low GABA and the magnitude of potentiation in the presence of NS-1738 or PAM-2. In five cells, 50 µM NS-1738 potentiated the response to low GABA to 253 ± 72% of the control before exposure to pCMB and to 249 ± 57% of the control after exposure to pCMB. The P_A_ of the response to low (2–3 µM) GABA was 0.11 ± 0.08 before and 0.09 ± 0.02 after exposure to pCMB. In a different set of five cells, 50 µM PAM-2 potentiated the response to GABA to 154 ± 15% of the control before exposure to pCMB and to 157 ± 18% of the control after exposure to pCMB. The P_A_ of the responses to low (1–3 µM) GABA were 0.16 ± 0.10 and 0.14 ± 0.08 before and after pCMB, respectively. We infer that pCMB does not functionally modify native cysteines in the α1β2γ2L receptor.

### 3.4. Mutations to the Combinations of α+/β−, γ+/β−, and α+/γ− Interfaces Affect Receptor Activation and Potentiation by α7-PAMs

Mutations unique to the α+/β− and γ+/β− interfaces can be made by mutating the “−” side (TM1 domain) of the β2 subunit. Specifically, we tested the effects of the L223W, Q224A, and M227W mutations on the β2 subunit. In addition, we mutated the L275, T277, T281, and F304 residues at the “+” side of the γ2L subunit. These residues are unique to the γ+/β− interface. At the “+” side of the α1 subunit, we mutated the T264, T266, W287, A290, and Y293 residues, which introduced mutations to the α+/β− interface and, additionally, to the α+/γ− interface.

At the “−” side of the β2 subunit, potentiation by NS-1738 was not affected by any of the tested mutations. PAM-2 inhibited responses to GABA in β2(L223W), reducing the response to low GABA (P_A_ = 0.26 ± 0.13, *n* = 5) to 48% of the control. The constitutively active (P_A,constitutive_ = 0.15 ± 0.07, *n* = 10) γ2L(F304C) mutation to the γ+/β− interface converted NS-1738 (34% of the control) and PAM-2 (40% of the control) into inhibitory compounds, while γ2L(T277I) (P_A,constitutive_ = 0.08 ± 0.05, *n* = 10) increased potentiation by NS-1738 (511% of the control) but reduced potentiation by PAM-2 (117% of the control) (Table 1). 

At the “+” side of the α1 subunit (mutations to α+/β− and α+/γ− interfaces), the α1(Y293C) mutation turned NS-1738 into an inhibitory compound (62% of the control) and reduced the potentiating effect of PAM-2 (122% of the control). The α1(Y293F) mutation did not affect the actions of either drug (Table 1), which is suggestive of continued aromatic interactions between the α7-PAMs and the α1(Y/F293) residue. We conclude that the α+/β−, γ+/β−, and/or α+/γ− interfaces contribute to GABA_A_ receptor potentiation by NS-1738 and PAM-2.

### 3.5. Mutations to the α+/γ− Interface Affect Receptor Activation and Potentiation by α7-PAMs

Mutations to the “+” side of the α1 subunit described above do not allow distinction between the α+/β− and α+/γ− interfaces. To mutate solely the α+/γ− interface, we tested the effects of mutations on the “−” side of the γ2L subunit. The γ2L(I242) residue was mutated to a serine and a tryptophan, and the γ2L(L246) residue was mutated to an asparagine and a tryptophan. The γ2L(I242W) and γ2L(I242S) mutations were without effect on the potentiating actions of NS-1738 and PAM-2 (Table 1). The γ2(L246N) and γ2L(L246W) mutations, which showed considerable constitutive activity (P_A,const_ = 0.25 ± 0.05 and 0.07 ± 0.04, respectively), were tested for direct activation by NS-1738 and PAM-2. Constitutive activity in the γ2(L246N)-containing receptor was inhibited by NS-1738 to 40% of the control and by PAM-2 to 75% of the control. In the γ2(L246W)-containing receptor, constitutive activity was enhanced to 150% of the control in the presence of NS-1738 and to 115% of the control in the presence of PAM-2. The calculated *c*_α7-PAM_ and ΔG values are given in Table 1. Overall, we conclude that the α+/γ− interface contributes to the actions of α7-PAMs.

### 3.6. Effects of Mutations to Intersubunit Interfaces on Receptor Activation and Potentiation by Selected Anesthetics

The high-affinity binding sites for the anesthetics propofol and etomidate are located in the transmembrane domain at the β+/α− intersubunit interfaces. Additionally, propofol binds to homologous pockets at the α+/β− and γ+/β− interfaces [23,34,35]. Potentiating neuroactive steroids make a major contribution through a site near the cytosolic end of the transmembrane helices at the β+/α− interface [36,37]. 

**Figure 6:**
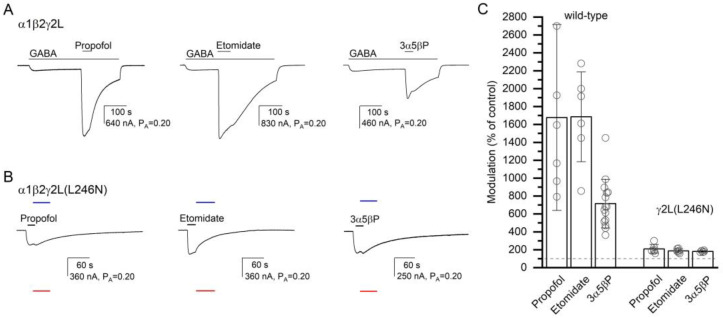
The modulatory effects of propofol, etomidate, and 3α5βP. The current traces show the potentiating effects of 50 µM propofol, 50 µM etomidate, or 10 µM 3α5βP in the α1β2γ2L receptor activated by 2–4 µM GABA (**A**) or the direct-activating effects of the compounds in the α1β2γ2L(L246N) receptor (**B**). The blue and red lines show the current levels in the same cell in the presence of 200 µM picrotoxin (assumed P_A_ = 0) or 1 mM GABA + 50 µM propofol (assumed P_A_ = 1), respectively. The modulatory effects are summarized in the column graph (**C**). The effects of the compounds are significantly reduced in the mutant receptor. In (**A**), modulation is calculated as the amplitude of the peak response to GABA + α7-PAM divided by the amplitude of the response to GABA alone at the time of peak response to GABA + α7-PAM. In (**B**), modulation is calculated as the ratio of the estimated P_A_ in the presence and absence of α7-PAM. Drug application durations are given with horizontal lines.

To gain insight into the specificity of the effect of the γ2L(L246N) (α+/γ− interface) mutation on receptor activation by α7-PAMs, we recorded activation of the α1β2γ2L(L246N) receptor by propofol, etomidate, or 3α5βP. Previous work has suggested that the anesthetics or a neuroactive steroid do not interact with the α+/γ− interface [30]. In six cells exposed to propofol and etomidate, and for normalization purposes to 1 mM GABA + 50 µM propofol and 200 µM picrotoxin, 50 µM propofol or etomidate increased channel activity from a P_A,constitutive_ of 0.33 ± 0.07 to a P_A_ of 0.69 ± 0.16 (209% of the control; S.D. and numbers of oocytes are given in Table 2) or 0.63 ± 0.17 (187% of the control), respectively. With the postulated numbers of binding sites of four for propofol and two for etomidate, the calculated *c*_50 µM drug_ are 0.677 (ΔG = −0.96 kcal/mol) and 0.525 (ΔG = −0.87 kcal/mol) for propofol and etomidate, respectively. For comparison, in the α1β2γ2L receptor, 50 µM propofol increased the response to 1–2 µM GABA (P_A_ = 0.07 ± 0.04; *n* = 6) to 1677% of the control (P_A,50 µM propofol_ = 0.86 ± 0.08, *c*_50 µM propofol_ = 0.302, ΔG = −2.84 kcal/mol), and co-application of 50 µM etomidate increased the response to low GABA (P_A_ = 0.05 ± 0.02, *n* = 6) to 1685% of the control (P_A,50 µM etomidate_ = 0.75 ± 0.06, *c*_50 µM etomidate_ = 0.129, ΔG = −2.45 kcal/mol). The ΔG values for both propofol and etomidate are statistically significantly different (*t*-test, *p* < 0.001 for each comparison) in α1β2γ2L and the γ2(L246N) mutant. Sample current traces and data summary are given in Figure 6 and Table 2.

We also recorded receptor activation and potentiation by the steroid 3α5βP. In five cells expressing α1β2γ2L(L246N) receptors, the application of 10 µM 3α5βP increased the P_A_ from 0.29 ± 0.06 to 0.53 ± 0.11 (181% of the control). Sample current traces are given in Figure 6. The calculated *c*_10 µM 3α5βP_ is 0.604, and the ΔG is −0.60 kcal/mol. For comparison, in the α1β2γ2L wild-type receptor, co-application of 10 µM 3α5βP increased the response to low GABA (P_A_ = 0.07 ± 0.04, *n* = 15) to 715% of the control (*c*_10 µM 3α5βP_ = 0.303 and ΔG = −1.45 kcal/mol). The effects are statistically significantly different (*t*-test, *p* < 0.001). In sum, we infer that the γ2L(L246N) mutation at the α+/γ− interface disrupts the potentiating/activating effects of propofol, etomidate, and 3α5βP. 

To determine if other intersubunit interfaces similarly influence the actions of 3α5βP, we recorded steroid-mediated potentiation or direct activation in receptors containing mutations to the anesthetic-binding interfaces. The β2(F289A) mutation at the β+/α− interface, and the γ2L(T277I) and γ2L(F304C) mutations at the γ+/β− interface, significantly reduced receptor activation by 3α5βP. The magnitudes of effects, however, were relatively small (Table 3), which is suggestive of a less prominent interplay between the anesthetic-binding interfaces and the neurosteroid binding sites.

### 3.7. The Effects of Mutations Indicate Lack of Additivity and Independence between Intersubunit Anesthetic Binding Sites

Results from mutated receptors indicate that mutations to each of the intersubunit interfaces affect potentiation by NS-1738 and PAM-2. To estimate the energetic additivity of the effects of mutations, we employed mutant cycle analysis in which the free energy change (ΔG_α7-PAM,WT_) in the wild-type receptor was compared with the sum of changes in free energy changes (ΔΔG_α7-PAM,MT_) in receptors containing mutations to individual interfaces. In the case of energetic additivity, ΔG = −∑ΔΔG_i_.

We selected the β2(F289T) mutation to the two β+/α− interfaces, the α1(Y293C) mutation to the α+/β− and α+/γ− interfaces, and the γ2L(F304C) mutation to the γ+/β− interface. For NS-1738, the ΔΔGs with 95% confidence intervals are 1.92 [1.50 to 2.34] kcal/mol, 1.01 [0.86 to 1.16] kcal/mol, and 1.44 [1.17 to 1.71] kcal/mol for mutations in β+/α−, α+/β− and α+/γ−, and γ+/β−, respectively. The sum of ΔΔGs is 4.37 [3.85 to 4.89] kcal/mol. The 95% confidence interval for −∑ΔΔG_i_ does not include the inverse of ΔG_NS-1738,WT_ (0.70 kcal/mol, Table 1), indicating that the effects of mutations are not additive or independent. Intuitively, this is evident from the observation that rather than the individual mutations incrementally reducing the potentiating effect of NS-1738 and the sum of effects of mutations predicting a complete loss of potentiation, each of the individual mutations actually leads to functional inhibition in the presence of NS-1738.

For PAM-2, the ΔΔGs with 95% confidence intervals are 0.44 [0.28 to 0.60] kcal/mol, 0.26 [0.21 to 0.31] kcal/mol, and 1.05 [0.80 to 1.30] kcal/mol for β2(F289T), α1(Y293C), and γ2L(F304C), respectively. The sum of ΔΔGs is 1.75 [1.45 to 2.05] kcal/mol. The 95% confidence interval for −∑ΔΔG_i_ does not include the inverse of ΔG_PAM-2,WT_ (0.39, Table 1), indicating that the effects of mutations are not additive or independent.

## 4. Discussion

Our molecular docking and molecular dynamics studies indicate that the α7-PAMs NS-1738 and PAM-2 bind to the intersubunit interfaces in the transmembrane domain of the GABA_A_ receptor. This is supported by previous functional data demonstrating a reduction or loss of potentiation by α7-PAMs of receptors activated by drugs binding to the anesthetic binding sites in the transmembrane domain [22]. In the present study, we have also shown that NS-1738 and PAM-2 protect against pCMB-induced chemical modification of the α1(L231C) residue at the β+/α− interface.

The major finding from our mutational analysis is that mutations to the putative binding sites at individual intersubunit interfaces can strongly alter the modulatory effect of α7-PAMs, with effects ranging from a significant increase in potentiation to inhibition of receptor function. This is surprising and unexpected if we assume that the mutations only act locally without modifying drug interactions at other interfaces or global receptor functions. With a few exceptions, the mutations similarly affected receptor potentiation by NS-1738 and PAM-2. The exceptions were β2(L223W) at the α+/β− and γ+/β− interfaces, which allowed for potentiation by NS-1738 but converted PAM-2 into an inhibitory compound, and α1(Y293C), which caused NS-1738 to inhibit receptor function but merely reduced the ability of PAM-2 to potentiate. We considered the possibility that α7-PAMs act as potentiators of the α1β2γ2L GABA_A_ receptor through one or more interfaces and as inhibitors through other interfaces. For example, the β2(F289T) mutant or the chemical modification of the α1(L231C) residue (both at the β+/α− interface) result in NS-1738 inhibiting receptor function, potentially suggesting that the β+/α− interface mediates potentiation whereas the remaining, intact interfaces mediate receptor inhibition. This, however, is contradicted by the observation that mutations to other interfaces (γ2L(F304C) to γ+/β−, γ2L(L246N) to α+/γ−, and α1(Y293C) to α+/β− and α+/γ− interfaces) could also reverse the polarity of effect. 

The data summarized in Table 1 indicate that the effects of mutations to individual interfaces are not local and independent, or, alternatively, that the binding sites for α7-PAMs are allosterically linked. In the case of local and independent action, individual mutations may be expected to have an incremental effect on overall potentiation, while complete loss of potentiation may be observed when there are no intact sites remaining. Such a scenario has been observed previously with mutations affecting GABA_A_ receptor activation by the transmitter or potentiation of receptor function by propofol, etomidate, or allopregnanolone [38,39,40,41]. Instead, we observed that a single mutation to any individual interface could fully abolish potentiation by either α7-PAM. Possible explanations for the findings are long-range effects of mutations and effects on the channel gating process shifting the equilibrium between resting and active states. The latter idea is supported by the finding that the γ2L(L246N) mutation additionally reduces the gating efficacy of the anesthetics propofol and etomidate and the neurosteroid 3α5βP, neither of which is expected to bind at the α+/γ− interface (Table 2). Furthermore, the tryptophan substitution of this residue reduces the gating efficacy of the etomidate and the transmitter GABA [30].

In previous work, mutations to anesthetic sites at the β+/α−, γ+/β−, and α+/β− interfaces modified receptor potentiation by the anesthetic drugs propofol, etomidate, and a barbiturate as well as activation by the transmitter GABA [19,30]. Similar to what we have observed with NS-1738 and PAM-2, the sum of losses of potentiating effects in individual mutants was consistently greater than the potentiating effect observed in the wild-type receptor, which is potentially indicative of allosteric crosstalk between the interfaces. GABA_A_ receptor potentiation by neuroactive steroids is mediated by two classes of membraneous binding sites, one at the β+/α− intersubunit interface and the other within the α subunit [37]. Mutations to the two classes of binding sites have been shown to demonstrate independence and additivity in the α1β2γ2L receptor and lack of it in the α1β3 receptor [41,42]. Mutation of either one of the two transmitter binding sites reduces gating efficacy for GABA approximately, equally, and independently [38].

The conclusions of this study ultimately arise from the comparison of the magnitude of potentiation at a single concentration of a compound. The concentrations of the α7-PAMs, anesthetics, and 3α5βP used are saturating in the wild-type receptor [14,15,18,22,43], and thus reflect maximal potentiation and a true value of *c*_compound_. There is, however, no definite evidence that the same concentrations of the compounds are saturating in the mutant receptors. Thus, the estimated *c*_compound_ in mutant receptors should be treated as “effective” *c*_compound_, while the changes in *c*_compound_ and ΔG presented in Table 1, Table 2 and Table 3 may be due to altered affinity, efficacy, or both.

PAMs of the α7 nicotinic receptor may be clinically useful in the treatment of cognitive symptoms in schizophrenia. In rodents, administration of α7-PAMs such as PNU-120596 or PAM-2 improves the auditory gating deficit caused by amphetamine and recognition memory and cognitive flexibility in the MK-801 model of schizophrenia [44,45,46]. The present, as well as prior [22], data indicate that α7-PAMs can potentiate several common subtypes of the GABA_A_ receptor through interactions with the classic anesthetic binding sites. However, given their relatively low efficacies, α7-PAMs are expected to competitively inhibit GABA_A_ receptor potentiation by propofol or etomidate and any resulting behavior.

## 5. Conclusions

The PAMs of the α7 nicotinic receptor, NS-1738 and PAM-2, potentiate the GABA_A_ receptor through interactions with the classic anesthetic binding sites. Mutations to each of the four classes of intersubunit interfaces in the transmembrane domain of the α1β2γ2L receptor can modify potentiation. Overall, there was good agreement between predictions made based on molecular docking studies, SCAMP, and mutational-functional analysis. Comparison of the magnitudes of effects of mutations to the individual intersubunit interfaces suggests that the sites mediating the actions of α7-PAMs are allosterically linked.

## Figures and Tables

**Table 1:** Summary of effects of mutations on GABA_A_ receptor potentiation or direct activation by the α7-PAMs NS-1738 and PAM-2.

Receptor	Modulation, 50 µM NS-1738, % of Control (*n*)	*c*_50 µM NS-1738_	ΔG_50 µM NS-1738_, kcal/mol	Modulation, 50 µM PAM-2, % of Control (*n*)	*c*_50 µM PAM-2_	ΔG_50 µM PAM-2_, kcal/mol
α1β2γ2L	316 ± 123 (25)	0.561 ± 0.106	−0.70 ± 0.23	189 ± 36 (25)	0.721 ± 0.061	−0.39 ± 0.10
β+/α− interface						
β2(V258M)	720 ± 140 (5)	0.307 ± 0.044	−1.40 ± 0.16 *	301 ± 87 (5)	0.559 ± 0.110	−0.70 ± 0.22 *
β2(T262V)	126 ± 12 (5)	0.883 ± 0.045	−0.15 ± 0.06 *	121 ± 9 (5)	0.906 ± 0.032	−0.12 ± 0.04 *
β2(F289T) ^#^	21 ± 14 (5)	2.980 ± 1.097	1.22 ± 0.47 *	98 ± 29 (8)	1.058 ± 0.206	0.05 ± 0.23 *
β2(F289A) ^#^	74 ± 55 (5)	1.525 ± 0.845	0.37 ± 0.60 *	87 ± 20 (5)	1.094 ± 0.123	0.10 ± 0.13 *
α1(I227W)	207 ± 56 (5)	0.769 ± 0.163	−0.33 ± 0.24	135 ± 18 (5)	0.856 ± 0.059	−0.19 ± 0.08 *
α1(L231C)	349 ± 133 (5)	0.532 ± 0.105	−0.76 ± 0.23	197 ± 19 (5)	0.691 ± 0.037	−0.44 ± 0.06
α1(M235W) ^#^	196 ± 46 (5)	0.713 ± 0.108	−0.41 ± 0.17	174 ± 20 (5)	0.749 ± 0.041	−0.34 ± 0.06
α+/β− and γ+/β− interfaces						
β2(L223W)	215 ± 26 (5)	0.635 ± 0.054	−0.54 ± 0.10	48 ± 10 (5)	1.540 ± 0.157	0.50 ± 0.13 *
β2(Q224A)	389 ± 182 (6)	0.492 ± 0.118	−0.87 ± 0.28	169 ± 26 (5)	0.752 ± 0.058	−0.34 ± 0.09
β2(M227C)	335 ± 62 (5)	0.533 ± 0.055	−0.75 ± 0.12	143 ± 18 (5)	0.833 ± 0.051	−0.22 ± 0.07
γ+/β− interface						
γ2L(L275C)	283 ± 66 (5)	0.569 ± 0.082	−0.67 ± 0.16	251 ± 21 (5)	0.611 ± 0.029	−0.58 ± 0.06 *
γ2L(T277I) ^#^	511 ± 178 (5)	0.375 ± 0.082	−1.18 ± 0.26 *	117 ± 6 (5)	0.919 ± 0.025	−0.10 ± 0.03 *
γ2L(T281I)	262 ± 61 (6)	0.592 ± 0.078	−0.63 ± 0.16	255 ± 40 (5)	0.687 ± 0.051	−0.45 ± 0.09
γ2L(F304C) ^#^	34 ± 17 (5)	1.917 ± 0.449	0.74 ± 0.29 *	40 ± 17 (5)	1.791 ± 0.428	0.66 ± 0.28 *
α+/γ− and α+/β− interfaces						
α1(T264V)	295 ± 63 (5)	0.530 ± 0.079	−0.76 ± 0.17	240 ± 51 (5)	0.620 ± 0.075	−0.57 ± 0.14
α1(T266M)	151 ± 39 (5)	0.189 ± 0.107	−0.24 ± 0.16 *	147 ± 66 (6)	0.855 ± 0.133	−0.20 ± 0.21 *
α1(W287A)	292 ± 81 (5)	0.584 ± 0.091	−0.65 ± 0.18	182 ± 57 (6)	0.747 ± 0.092	−0.35 ± 0.16
α1(A290C)	262 ± 41 (5)	0.596 ± 0.042	−0.61 ± 0.08	215 ± 45 (5)	0.659 ± 0.082	−0.50 ± 0.14
α1(Y293F)	311 ± 140 (5)	0.567 ± 0.133	−0.70 ± 0.29	184 ± 56 (5)	0.735 ± 0.095	−0.37 ± 0.16
α1(Y293C)	62 ± 14 (5)	1.310 ± 0.164	0.31 ± 0.14 *	122 ± 7 (6)	0.899 ± 0.025	−0.13 ± 0.03 *
α+/γ− interface						
γ2L(I242W)	314 ± 44 (5)	0.541 ± 0.045	−0.73 ± 0.10	166 ± 12 (5)	0.766 ± 0.030	−0.31 ± 0.05
γ2L(I242S)	376 ± 106 (6)	0.493 ± 0.099	−0.85 ± 0.21	174 ± 12 (5)	0.744 ± 0.031	−0.35 ± 0.05
γ2L(L246W) ^#^	150 ± 23 (5)	0.800 ± 0.081	−0.27 ± 0.12 *	115 ± 5 (5)	0.928 ± 0.022	−0.09 ± 0.03 *
γ2L(L246N) ^#^	40 ± 25 (5)	2.161 ± 1.150	0.80 ± 0.55 *	75 ± 5 (5)	1.205 ± 0.047	0.22 ± 0.05 *

The columns give receptor specifics, the modulatory effect (number of oocytes), and the calculated values of *c* and ΔG. All values are given as mean ± S.D. Modulation is expressed in % of the control response (100% = no effect) to low GABA. For receptors with P_A,constitutive_ > 0.02 (marked with ^#^), the effects of α7-PAMs were measured in the absence of GABA as an effect on holding current, and modulation is calculated as P_A,α7-PAM_/P_A,constitutive_. *c*, the ratio of the equilibrium dissociation constants in active and resting receptors, is a measure of gating efficacy. A value less than one indicates that the compound is an activator. ΔG (in kcal/mol) expresses the free energy change contributed by the compound. A negative value indicates that the compound stabilizes the active state. Statistical significance between the effects of α7-PAMs in wild-type and mutant receptors was determined by one-way ANOVA (NS-1738: F(24,124) = 32.11, *p* < 0.001; PAM-2: F(24,126) = 33.77, *p* < 0.001), and followed by Dunnett’s post-hoc multiple comparisons test (*, *p* < 0.05).

**Table 2:** Summary of effects of the γ2(L246N) mutation to the α+/γ− interface on GABA_A_ receptor potentiation or direct activation by propofol, etomidate, and 3α5βP.

Receptor	Modulation, 50 µM Propofol, % of Control (*n*)	*c*_50 µM propofol_	ΔG_50 µM propofol_, kcal/mol	Modulation, 50 µM Etomidate, % of Control (*n*)	*c*_50 µM etomidate_	ΔG_50 µM etomidate_, kcal/mol	Modulation, 10 µM 3α5βP, % of Control (*n*)	*c*_10 µM 3α5βP_	ΔG_10 µM 3α5βP_, kcal/mol
α1β2γ2L	1677 ± 1039 (6)	0.302 ± 0.039	−2.84 ± 0.33	1685 ± 501 (6)	0.129 ± 0.030	−2.45 ± 0.27	715 ± 271 (15)	0.303 ± 0.069	−1.45 ± 0.34
α+/γ− interface									
γ2L(L246N) ^#^	209 ± 49 (6)	0.677 ± 0.122	−0.96 ± 0.45 *	187 ± 22 (6)	0.525 ± 0.184	−0.87 ± 0.63	181 ± 9 (5)	0.604 ± 0.050	−0.60 ± 0.10

The columns give receptor specifics, the modulatory effect (number of oocytes), and the calculated values of *c* and ΔG. All values are given as mean ± S.D. Modulation is expressed in % of the control response (100% = no effect) to low GABA in α1β2γ2L. For the constitutively active (^#^) γ2(L246N)-containing receptor, the effects were measured in the absence of GABA as an effect on holding current, and modulation is calculated as P_A,compound_/P_A,constitutive_. *c*, the ratio of the equilibrium dissociation constants in active and resting receptors, is a measure of gating efficacy. A value less than one indicates that the compound is an activator. The number of imposed binding sites was four for propofol and two for etomidate and 3α5βP. ΔG expresses the free energy change contributed by the compound. A negative value indicates that the compound stabilizes the active state. Statistical significance between the effects of the compounds in wild-type and mutant receptors was determined by *t*-test (*, *p* < 0.001).

**Table 3:** Summary of effects of mutations on GABA_A_ receptor potentiation or direct activation by 3α5βP.

Receptor	Modulation, 10 µM 3α5βP, % of Control (*n*)	*c*_10 µM 3α5βP_	ΔG_10 µM 3α5βP_, kcal/mol
α1β2γ2L	733 ± 183 (15)	0.330 ± 0.038	−1.31 ± 0.14
β+/α− interface			
β2(F289A) ^#^	463 ± 118 (5)	0.453 ± 0.043	−0.94 ± 0.12 *
α1(M235W) ^#^	614 ± 116 (6)	0.304 ± 0.046	−1.42 ± 0.19
γ+/β− interface			
γ2L(T277I) ^#^	471 ± 262 (5)	0.473 ± 0.110	−0.91 ± 0.29 *
γ2L(F304C) ^#^	255 ± 31 (6)	0.493 ± 0.091	−0.86 ± 0.26 *
α+/β− and γ+/β− interfaces			
β2(L223W) ^#^	653 ± 186 (5)	0.298 ± 0.057	−1.45 ± 0.22
α+/γ− and α+/β− interfaces			
α1(Y293C)	666 ± 209 (5)	0.266 ± 0.055	−1.58 ± 0.23
α+/γ− interface			
γ2L(L246N) ^#^	181 ± 9 (5)	0.604 ± 0.050	−0.60 ± 0.10 *

The columns give receptor specifics, the modulatory effect (number of oocytes), and the calculated values of *c* and ΔG. All values are given as mean ± S.D. Modulation is expressed in % of the control response (100% = no effect) to low GABA. For receptors with P_A,constitutive_ > 0.02 (marked with ^#^), the effect of 3α5βP was measured in the absence of GABA as an effect on holding current, and modulation is calculated as P_A,α7-PAM_/P_A,constitutive_. *c*, the ratio of the equilibrium dissociation constants in active and resting receptors, is a measure of gating efficacy. A value less than one indicates that the compound is an activator. The number of imposed steroid binding sites was two. ΔG (in kcal/mol) expresses the free energy change contributed by the compound. A negative value indicates that the compound stabilizes the active state. Statistical significance between the effects of α7-PAMs in wild-type and mutant receptors was determined by one-way ANOVA (F(7,44) = 11.43, *p* < 0.001), and followed by Dunnett’s post-hoc multiple comparisons test (*, *p* < 0.01). Data on γ2L(L246N) is replicated from Table 2.

## Data Availability

The data presented in this study are available on request from the corresponding author.

## Supplementary Materials

The following supporting information can be downloaded at: https://www.mdpi.com/article/10.3390/biom13040698/s1, pdb structures: PDB files showing docked NS-1738 and PAM-2 at each of the four interfaces (β+/α−, α+/β−, α+/γ−, and γ+/β−) in the α1β2γ2 GABA_A_ receptor.

## Abbreviations

3α5βP	5β-pregnan-3α-ol-20-one
*c*	ratio of the equilibrium dissociation constant in the active state to that in the resting state (a measure of efficacy)
ΔG	free energy change
GABA	γ-aminobutyric acid
GABA_A_	γ-aminobutyric acid type A
K_R_	equilibrium dissociation constant of a compound in the resting receptor
NS-1738	*N*-(5-Cl-2-hydroxyphenyl)-*N*′-[2-Cl-5-(trifluoromethyl)phenyl]-urea
P_A_	probability of being in the active state;
PAM	positive allosteric modulator
PAM-2	(*E*)-3-(furan-2-yl)-*N*-(*p*-tolyl)-acrylamide.
